# Supplementary material for: Branched amphotericin functional poly(N-isopropyl acrylamide): an antifungal polymer
Source: R Soc Open Sci. 2021 Jan 13;8(1):201655. doi: 10.1098/rsos.201655 (PMC7890487; doi:10.1098/rsos.201655)
Supplement: supplementary material [file rsos201655supp1.docx]

**Branched Amphotericin Functional Poly(N-*iso*propyl acrylamide): an Antifungal Polymer**

**Thomas Swift, Emily Caseley, Abbigail Pinnock, Joanna Shepherd, Nagaveni Shivshetty, Prashant Garg, C. W. Ian Douglas, Sheila MacNeil and Stephen Rimmer**

Supplementary Materials:

1. Materials

All materials were used as supplied unless otherwise stated. Stated material purities were N-*iso*propyl acrylamide (NIPAM) (Adrich, 97%), N-hydroxy succinimide (Aldrich, 98%), dicyclohexyl carbodiimide (Aldrich, 98%), Amphotericin B (Cambridge Bioscience, >95%), dichloromethane (HPLC grade), Nile red (Sigma, 99%) and ethyl acetate (HPLC grade) were supplied by Sigma Aldrich. N-isopropyl acrylamide was recrystallized from hexane/toluene. 4,4’-Azobis(4-cyanovaleric acid) (ACVA) (98% purity) was obtained from Alfa Aeser and dried in vacuo at room temperature overnight. Branching chain transfer agent (CTA) 4-Vinylbenzyl- pyrrole-carbodithioate (**1**) was synthesized and purified according to a previously reported method.(*1*) Dioxane (Analar grade), diethyl ether (anhydrous), dimethyl formamide (Analar grade), ethanol (Analar grade), acetone (Analar grade) and hydrochloric acid (35% wt/wt) were obtained from VWR, Ltd. Cell culture reagents were purchased from Sigma-Aldrich unless otherwise stated. Dispase II was purchased from Roche and epidermal growth factor was purchased from Invitrogen. Mouse 3T3 fibroblasts were obtained from ATCC, Manassas, VA and an established cell line-J2 3T3 cells originally obtained from Professor Howard Green, USA. Wild brown rabbit heads were kindly provided by the BlackFace Meat Company in Dumfries, Scotland. Human corneas were obtained from Ramayamma International Eye Bank, LV Prasad Eye Institute, Hyderabad. Due to the low solubility of Amphotericin B(*2*), for biological assays, Amphotericin B BioReagent (Sigma-Aldrich) was used.

**2.** *Synthesis of HB-PNIPAM-COOH.* HB-PNIPAM with carbodithioate end groups (HB-PNIPAM-Py) was prepared via dissolution of **1** (3.667 g, 14.1 mmol), ACVA (3.96 g, 14. 1 mmol) and NIPAM (40.00 g, 353.5 mmol) in 1,4-dioxane (280 mL) in a sealed round bottom flask. This was bubbled with nitrogen for thirty minutes to degas the solvent before heating (60 ˚C, 48 hours) under a nitrogen atmosphere. The reaction mixture was precipitated into diethyl ether and the precipitate collected and dried in vacuo overnight. The solid was weighed and characterized by ^1^H NMR [400 MHz, DMSO (ppm): 1.02 (6H, s, -N(CH_3_)_2_) 1.40 (2H, br m, CH_2_-CH-Ar-) 1.95 (2H, br m, -CH_2_-CH-CO-NH-), 3.82 (H_2_O-polymer), 6.41 (H_2_, br s, N-pyrrole-H), 7.13 (br m, -Ar-), 7.7 (2H br s, N-pyrrole-H)], Sulphur content 1.81 %, T_c-g_ = 19.0 °C and) R_Hn_ = 2.33, R_Hw_ = 2.40 (DOSY (DMSO) 298 K).

The HB-PNIPAM-Py was dissolved (5.0 g) in 400 mL dimethyl formamide with ACVA (73.22 g, 259.2 mmol), nitrogen was bubbled through the solution which was then heated and stirred for 18 hours. Additional ACVA was added to the mixture (73.22 g, 259.2 mmol) which was reacted for an additional 18 hours before a final addition of ACVA (73.22 g, 259.2 mmol) and further reacted for another final 18 hours. The reaction mixture was precipitated into diethyl ether then reprecipitated from acetone into diethyl ether. The final product was dissolved in acetone with 10% ethanol and ultrafiltered three times to remove the low molar mass component, then the solvent was removed by rotary evaporation. The final solid yield of HB-PNIPAM-COOH was 95 % ^1^H NMR [400 MHz, DMSO (ppm): 1.02 (6H, s, -N(CH_3_)_2_) 1.43 (2H, br m, CH_2_-CH-Ar-) 1.98 (2H, br m, -CH_2_-CH-CO-NH-), 3.79 (H_2_O-polymer), 7.17 (br m, -Ar-)], Sulphur content = 0.25 %, T_c-g_ = 22.2 °C. The polymer was stored at -18 ºC.

3. Instrumentation

Polymer characterisation was carried out by nuclear magnetic resonance (NMR) spectroscopy of solutions in DMSO-D_6_. Measurements were obtained with a Bruker Avance spectrometer operating at 400 MHz (^1^H) and 600 MHZ (^13^C). The sulphur content was determined by elemental analysis via combustion as % of total sample by weight. LCST measurements were carried out on a Nano DSC by TA Instruments; polymer samples were dissolved in H_2_O at 5 mg ml^-1^ and stored at 5 °C for 24 hours prior to use to ensure complete dissolution. Samples were run over the temperature range 3–75 °C with a heating rate of 1.5 °C per minute and cooling rate of 1 °C per minute. The T_c–g_ was taken as the temperature at the thermogram peak maximum. Electrospray mass spectra of the polymer were recorded using a Micromax Quattro LC from Kinesis Solutions. UV absorbance measurements were carried out both in-line on the GPC instrumentation and in 1 cm path length cuvettes using a Varian Cary 50 probe UV-Visible spectrometer.

Polymer molar mass distribution was recorded via gel permeation chromatography (GPC) on a methanol based system as we described previously.(*3*) Samples were dissolved in methanol (1 mg mL^-1^) and injected (200 μL) through two Agilent Polargell-M columns (high molar mass range) with a flow rate of 1 mL min^-1^. They were analysed via comparison to a universal calibration using linear PNIPAM standards via triple RI, UV and viscometric detection to give absolute molar mass averages (M_n_, M_w_ M_z_).

The polymer hydrodynamic radius was calculated via DOSY NMR spectroscopy to calculate diffusion (D), which was converted to hydrodynamic radius using the Stokes Einstein equation as shown in equation 1. Sample internal viscosity (η) was found using sample solvent shift as outlined previously.^3^

$D= \frac{kT}{6\pi\eta R}$ Equation 1

**4. Purity of Amphotericin B**

**4.1 LC-MS**


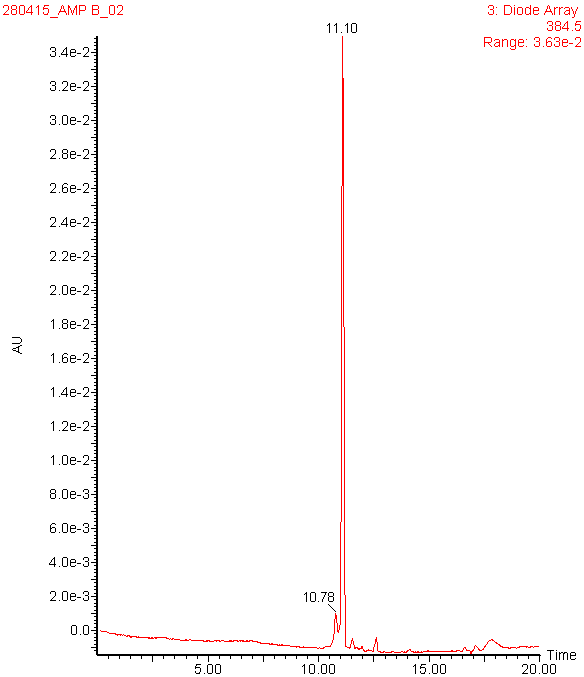


**Figure S1** HPLC (UV)-AmB


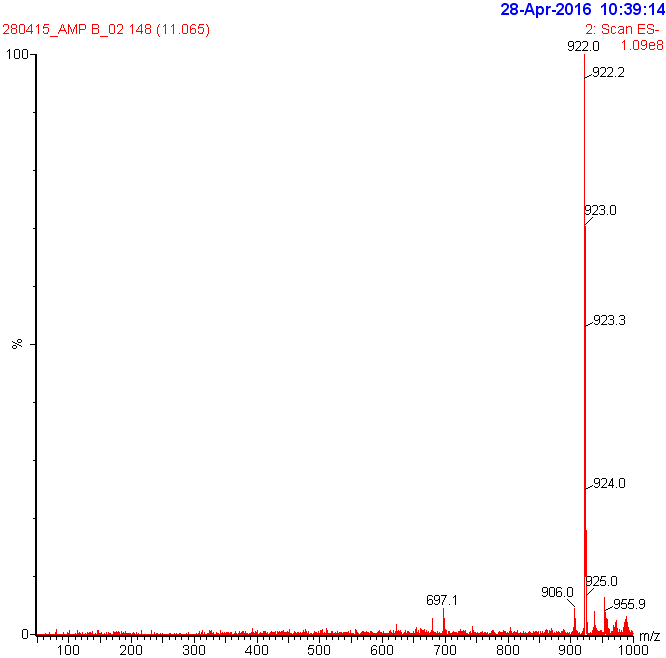


**Figure S2** Mass spectrum of sample eluting at 11.10 minutes


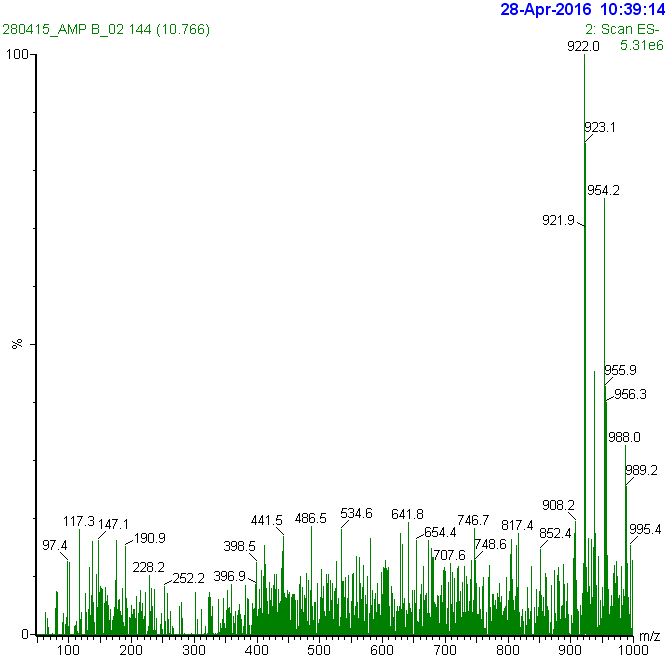


**Figure S3** Mass of Sample eluting at 10.78 minutes

# **4.2** Calibration of Mass Spectrometry Absorption

Figure S4 shows the calibration curve for AmB obtained from electrospray mass spectrometry carried out in methanol. This experiment was repeated in the presence of non-functionalised polymer and a small reduction in sensitivity was noted, although all solutions above 0.27 μmol dm^-3^ showed a strong peak from the molecular ion of AmB (m/z 946 in positive mode, m/z 922 in negative mode). The data showed that the limit of detection was 0.27 μmol dm^-3^ of AmB in methanol. The data were used to show that there was negligible residual AmB present after purification.


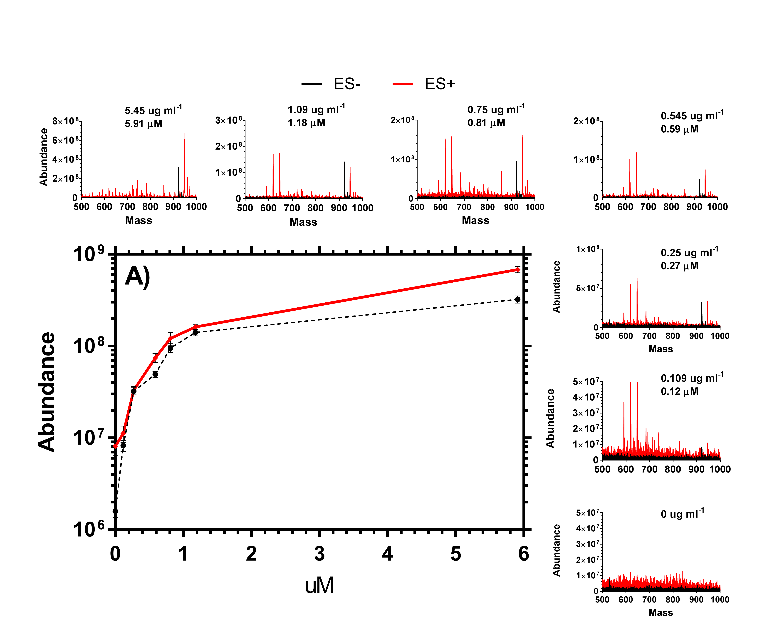


**Figure S4** Mass Spectrometry Average abundance response of detector to amphotericin injection (n = 3). Border insets are raw data following direct injection. ES- (black) and ES+ (red) shown

5. NMR

5.1 1H NMR

**Figure S5** ^1^H NMR spectrum of HB-PNIPAM-AmB polymer with expansion on 4-6 ppm region (inset, with non-functionalised HB-PNIPAM-Py provided for reference (blue))

**5.2 DOSY Measurements of Polymer Hydrodynamic Radius**


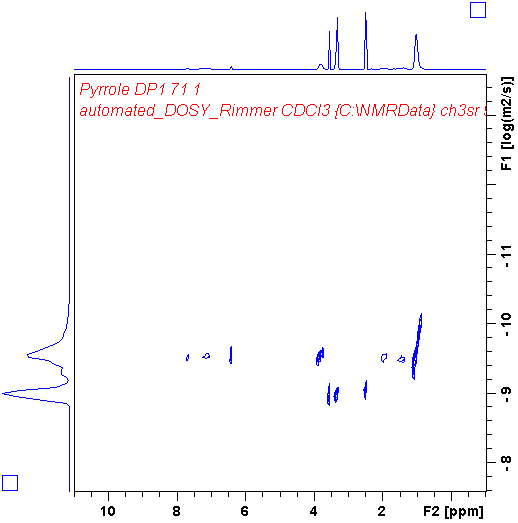


**Figure S6** Diffusion spectrum of HB-PNIPAM-Py.
Log D solvent -8.990, log D polymer -9.550


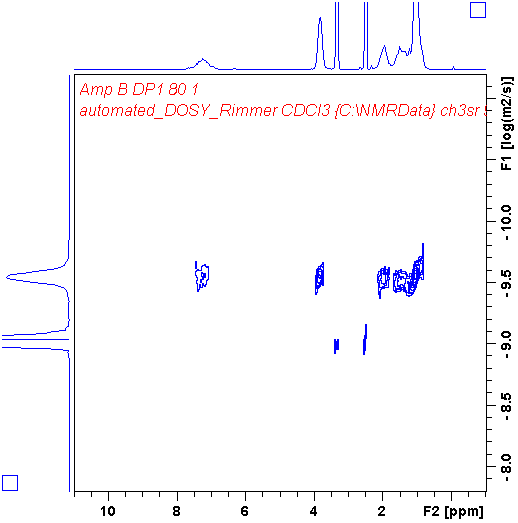


**Figure S7** Diffusion spectrum of HB-PNIPAM-AmB.
Log D solvent - -8.993, log D polymer -9.548

**6.** **Calorimetry**

**Figure S8** Thermogram micro-DSC data of HB-PNIPAM-Py (blue) and HB-PNIPAM-AmB (black). ------- HB-PNIPAM-Py, HB-PNIPAM-AmB

**7. Desolvation of polymer in presence of Nile red**

The collapse of highly branched polymers such as PNIPAM can be monitored using the addition of solvatochromic fluorescence dyes such as Nile red(*4*). The Nile red probe reports the internal environment (polarity) of the polymer coil(*4*). As the polymer chain was heated the emission wavelength decreased from 641 to 627 nm across 10 to 45 ^o^C (Figure 2, main manuscript). The data showing the shift in peak wavelength and intensity are shown in Figure S9.

**
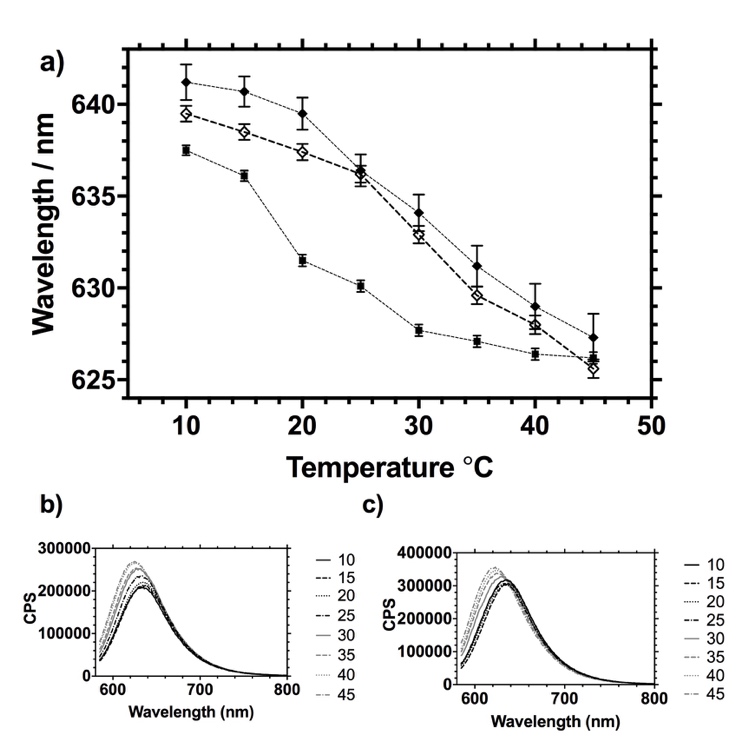
A B**

**Figure S9** Emission spectra at various temperatures in aqueous media **A)** HB-PNIPAM-AmB; **B)** HB-PNIPAM-AmB plus ergosterol

## **8. Measurement of inflammatory cytokine release**

Measurement of tumour necrosis factor alpha (TNF-α) and interleukin-1 beta (IL-1β) in cell culture media was analysed by sandwich enzyme-linked immunosorbent assay (ELISA) (Diaclone). PBMCs were plated at a density of 20,000 cells per well in 200 μL media in flat bottomed 96-well plates and incubated with polymers at the indicated concentrations for 3, 6, 12, 18 or 24 hr, after which the supernatant was retrieved and the assay carried out according to the manufacturer’s instructions. In brief, Nunc MaxiSorp 96 well plates were coated with 100 μL of diluted capture antibody and incubated at 4 ⁰C overnight. Wells were subsequently washed twice with 300 μL washing solution, and 100 μL blocking buffer added to each well and the plate incubated at room temperature (RT) (18 to 25°C) for 2 hours. Wells were washed three times as described above, following which 100 μL of each sample, standard, control and zero (cell culture media) was added in duplicate to wells. 50 μL of diluted biotinylated anti-TNF-α or anti-IL-1β antibody was added to each well, and the plate incubated at RT for 3 hours. The plate was washed twice, then 100 μL of Streptavidin-HRP solution was added to all wells and the plate incubated at RT for 30 minutes. The plate was washed twice, followed by the addition of 100 μL TMB substrate solution to all wells and its incubation at RT in the dark. Once the colour had developed, 100 μL H_2_SO_4_ stop reagent was added and the absorbance immediately read (450 nm primary wavelength, 630 nm reference wavelength) with a Tecan Infinite F50 plate reader running Magellan for F50 software.

**ESI References**

1. S. Hopkins, S. R. Carter, J. W. Haycock, N. J. Fullwood, S. MacNeil, S. Rimmer, Sub-micron poly(N-isopropylacrylamide) particles as temperature responsive vehicles for the detachment and delivery of human cells. *Soft Matter* **5**, 4928-4937 (2009).

2. A. Lemke, A. F. Kiderlen, O. Kayser, Amphotericin B. *Applied Microbiology and Biotechnology* **68**, 151-162 (2005).

3. T. Swift, R. Hoskins, R. Telford, R. Plenderleith, D. Pownall, S. Rimmer, Analysis Using Size Exclusion Chromatography of poly(N-isopropyl acrylamide) using Methanol as an Eluent. *Journal of Chromatography A* **1508**, 16-23 (2017).

4. R. Plenderleith, T. Swift, S. Rimmer, Highly-branched poly(N-isopropyl acrylamide)s with core-shell morphology below the lower critical solution temperature. *RSC Advances* **4**, 50932-50937 (2014).
